# Remarkable Responses to Chemoimmunotherapy in Ultra‐Rare Urachal Cancer: A Case Series and Reverse Translational Research

**DOI:** 10.1002/mco2.70516

**Published:** 2025-12-08

**Authors:** Hiroshi Kotani, Shigeki Sato, Kazuyoshi Shigehara, Daisuke Saito, Hiroyuki Sakaguchi, Akihiro Nishiyama, Isao Matsumoto, Atsushi Mizokami, Seiji Yano, Hiroaki Taniguchi

**Affiliations:** ^1^ Kanazawa University Hospital Kanazawa Ishikawa Japan

Dear Editor

1

Urachal cancer (URCA) is an exceedingly rare and aggressive type of bladder cancer involving the urachus, a fibrous remnant of the allantois that extends from the bladder to the umbilicus. URCA accounts for less than 1% of all bladder‐associated malignancies, with an estimated annual incidence of approximately 1 per 5,000,000 [1]. Prognosis is poor as URCA is often diagnosed at advanced stages, and no systemic therapies are approved for patients worldwide. Previous studies suggest that 5‐fluorouracil (5‐FU) plus platinum‐based chemotherapies or immune checkpoint blockade (ICB) may be effective in treating URCA. Histopathological features partially resemble those of gastric cancer (GC) or colorectal carcinoma (CRC), both commonly treated with 5‐FU and platinum‐based chemotherapies.

Histopathological analysis of the first patient in this study revealed a tumor subtype of signet‐ring cell carcinoma, which is more commonly observed in GC than in CRC [2]. Based on these findings, we hypothesized that the ATTRACTION‐4 regimens using combination therapy comprising S‐1, oxaliplatin, and nivolumab (SOX/nivo) may benefit URCA patients.

The first case was a 69‐year‐old female who presented with abdominal bloating. She had been diagnosed with stage IIIA URCA, confirmed by transurethral biopsy and imaging, and underwent standard surgery at the time of diagnosis 4 years ago. She was later confirmed to have URCA relapse based on tissue biopsies of multiple small peritoneal nodules and cytology of ascites. Histopathological analysis revealed that the tumor contained a type of signet‐ring cell carcinoma, which is relatively more common in GC compared to CRC, prompting treatment with one of the regimens for GC: SOX/nivo (Supporting Informaton). Since there were no measurable lesions according to RECIST v1.1, ascites and CA125 levels were evaluated as clinical responses without drainage during SOX/nivo therapy. A clear clinical response was observed at Week 6, which persisted until Week 39 and was considered a significant clinical benefit, as evidenced by the disappearance of ascites on CT and decreased CA125 levels (Figure 1Ai). Over the course of treatment, oxaliplatin and S‐1 were administered for a total of five cycles and eight cycles, respectively. Unfortunately, disease progression was confirmed by a significant increase in ascites at Week 45. SOX/nivo therapy was discontinued at Week 47, at which point she received two cycles of subsequent chemotherapy with platinum plus gemcitabine but showed no response. She passed away at Week 75 after the initiation of SOX/nivo.

**FIGURE 1:**
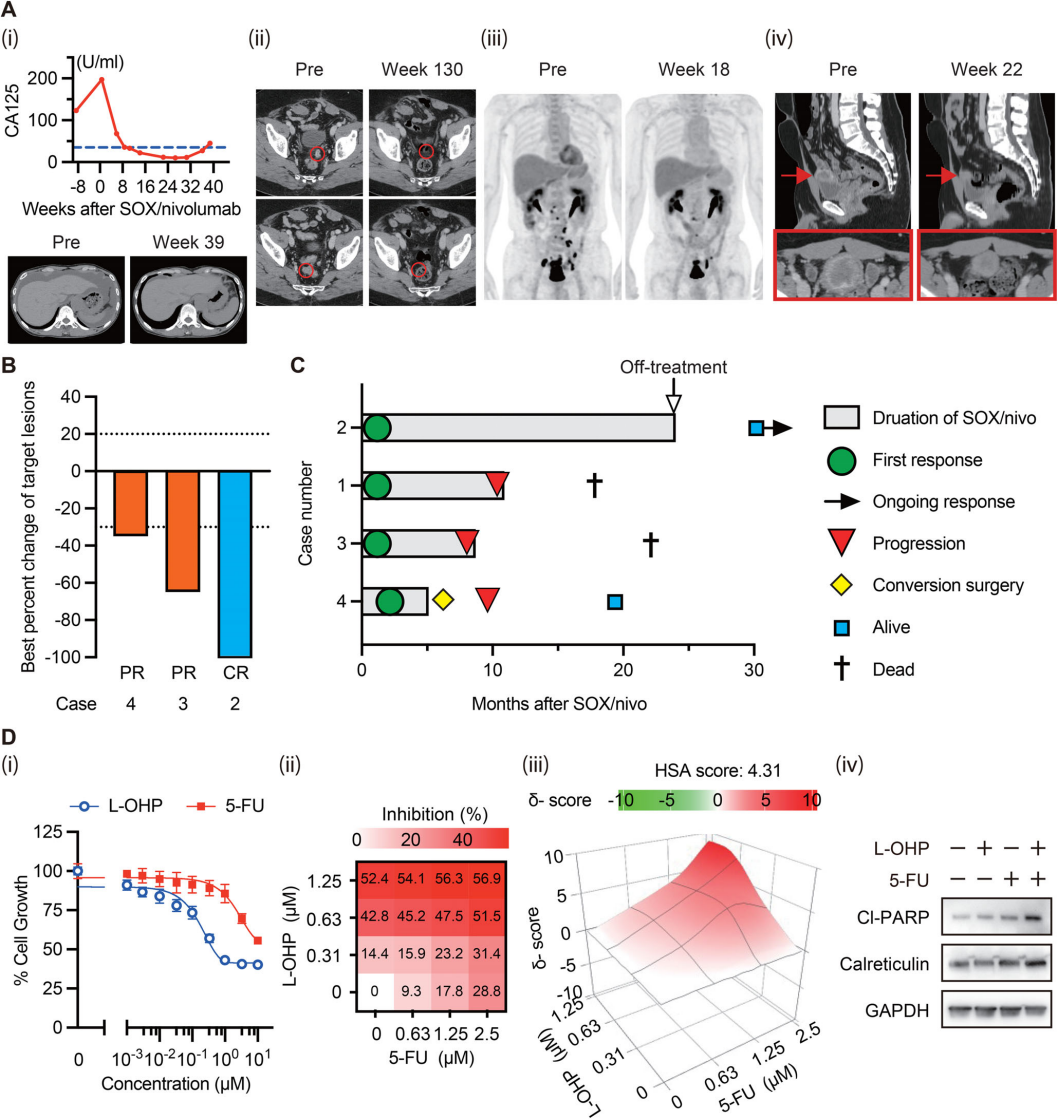
Remarkable response to SOX/Nivolumab in patients with urachal cancer and reverse translational research. (A) (i) Case 1 achieved a significant clinical benefit. After treatment, malignant ascites confirmed by cytology diminished without drainage, and the tumor marker CA125 markedly decreased. Representative CT images were taken before treatment and at Week 39. SOX/nivo: S‐1, oxaliplatin, and nivolumab therapy. (ii) Case 2 achieved a complete and long‐lasting response. The measurable lesions were peritoneal metastases. Representative CT images were taken before treatment and at Week 130. (iii) Case 3 achieved a partial response. Representative PET images were taken before treatment and at Week 18, at which point 18F‐fluorodeoxyglucose (FDG) accumulation was markedly reduced. (iv) Case 4 achieved a partial response. Two metastatic lung lesions were resected for diagnosis, leaving only one unresectable primary tumor. After the primary tumor shrank following SOX/nivo treatment, conversion surgery was performed. Representative CT images were taken before treatment and at Week 22. The upper and lower images show the sagittal views and enlarged axial views of the primary tumor, respectively. (B) A waterfall plot showing the best percent change from baseline among patients with measurable lesions, as assessed by the investigator using RECIST v1.1 criteria. Case 1 was excluded due to the lack of measurable lesions. CR, complete response; PR, partial response. (C) A swimmer plot showing the clinical course, including time to response, duration of response, and other events, for all four cases. (D) (i) Efficacy assessment of oxaliplatin (L‐OHP) or 5‐fluorouracil (5‐FU) in the urachal carcinoma cell line MISB18. (ii, iii) Combination effect of L‐OHP and 5‐FU in MISB18 cells. Dose‐response maps, including the highest single agent (HSA) score, were visualized using the SynergyFinder tool. (iv) Immunoblot analysis of MISB18 cells treated with L‐OHP (1 µM), 5‐FU (2 µM), or their combination for 24 h. Cl‐PARP, Cleaved poly ADP‐ribose polymerase; GAPDH, Glyceraldehyde‐3‐phosphate dehydrogenase.

The second case was a 56‐year‐old male who presented with hematuria. He had been diagnosed with stage IIIA URCA, confirmed by transurethral biopsy and imaging, and underwent standard surgery at the time of diagnosis 4 years ago. Two years after the surgery, he experienced local recurrence and underwent secondary surgery followed by five cycles of mFOLFOX6 at another hospital. He was later confirmed to have URCA relapse based on tissue biopsy of multiple peritoneal nodules and was subsequently treated with SOX/nivo. Although precautions were taken in advance to mitigate platinum‐induced allergies, given his treatment history, he developed severe anaphylaxis on Day 1 of two cycles of SOX/nivo, leading to the discontinuation of oxaliplatin. Despite this, a complete response (CR) was observed at Week 6, which was remarkable considering his prior treatment history. A representative response was shown by the CT images taken before treatment and at Week 130 (Figure 1Aii). CR persisted as of Week 130, with the last follow‐up 6 months after discontinuation of treatment.

The third case was a 58‐year‐old male who presented with hematuria. He was diagnosed with stage IVB URCA confirmed by transurethral biopsy and imaging. He underwent treatment with SOX/nivo and achieved a partial response (PR) at Week 6. A representative response was shown by FDG‐PET images before treatment and at Week 18 (Figure 1Aiii). Oxaliplatin was administered for the first six cycles but was stopped due to peripheral neuropathy, after which S‐1 plus nivolumab was continued. Disease progression was confirmed at Week 32, and treatment was discontinued at Week 37 due to the absence of further clinical benefit. He subsequently received several regimens commonly used for bladder cancer, but did not derive clinical benefit from any of them. He passed away at Week 95 after the initiation of SOX/nivo.

The fourth case was a 33‐year‐old male who presented with hematuria and was diagnosed with URCA by transurethral biopsy. FDG‐PET/CT imaging revealed the primary tumor with abnormal FDG accumulation, along with only two small lung nodules exhibiting faint FDG accumulations, which were suspected to be URCA‐associated lesions. Although a CT‐guided needle biopsy was attempted on one of the lung nodules to determine the stage of URCA, the targeted tissue could not be obtained due to the nodule's location and technical difficulties. Subsequently, both lung nodules were surgically resected and confirmed to be metastases of URCA. He underwent treatment with SOX/nivo and achieved a PR at Week 12. Following six cycles of SOX/nivo, conversion surgery was performed on the shrunken primary tumor. A representative response was demonstrated by CT images taken before treatment and at Week 22 (Figure 1Aiv). Although oligo‐lung metastasis recurred at Week 36 and other points, he remained alive as of Week 81, having undergone multiple CyberKnife treatments.

The best percent changes in their target lesions according to RECIST v1.1 and their clinical courses following the initiation of SOX/nivo are summarized in Figure 1B,C. Notably, all four patients achieved clear therapeutic benefits, despite the absence of programmed death‐ligand 1 (PD‐L1)‐high, microsatellite instability (MSI)‐high, or tumor mutational burden (TMB)‐high tumors. Additionally, no unexpected immune‐related adverse events occurred in these patients during SOX/nivo treatment.

We further conducted reverse translational research using a rare cell line, MISB18, derived from heavily treated patient with URCA (Supporting Informaton). MISB18 cells exhibited resistance to 5‐FU but showed modest sensitivity to oxaliplatin (Figure 1Di). Next, we evaluated the combinatorial efficacy of these agents. Interestingly, MISB18 cells remained sensitive to the combination therapy, demonstrating additive effects (Figure 1Dii,iii). Furthermore, the combination therapy induced apoptosis, as evidenced by elevated levels of cleaved‐PARP, and enhanced the antitumor immune response, as supported by increased calreticulin expression (Figure 1Div) [3].

We demonstrated, for the first time worldwide, the strong efficacy of SOX/nivo for unresectable or relapsed URCA. The response rates to 5‐FU and platinum‐based chemotherapies are approximately 40%, while the response rates to ICB are approximately 10% in URCA. Moreover, ICB monotherapies have been approved for use in GC, responding in 10%–20% but not in CRC, responding in less than 5%. Therefore, we considered that the therapeutic responses of URCA may align more closely with those of GC than with those of CRC, albeit with modest expectations. Although we cannot definitively conclude whether the efficacy of chemoimmunotherapy was additive or synergistic by cytotoxic agents with ICB, the precise mechanisms underlying the efficacy of chemotherapy combined with ICB remain unclear, even for more common tumors. Additionally, as mentioned earlier, the development of systemic therapies for URCA faces significant challenges due to its rarity. This is supported by the fact that 456 patients diagnosed with URCA for 6 years in total [4] (2008–2009 and 2012–2015), and 116 patients with URCA were registered in C‐CAT Japan nationwide for 5 years+ (2019–2024).

In conclusion, chemoimmunotherapy may represent promising treatment option for patients with unresectable or relapsed URCA. With caution regarding small studies’ limitations, we hope that drug development for rare malignancies will move forward.

## Author Contributions

H.K. contributed to the conceptualization, analysis, and interpretation of all data and writing the original draft. S.S., K.S., and D.S. contributed to the analysis and interpretation of the patient data. All the authors participated in patient care and approved the final manuscript.

## Funding

The authors have nothing to report.

## Ethics Statement

The treatments and clinical courses were reported to the Division of Medical Safety of Kanazawa University Hospital and approved by the Medical Ethics Committee of Kanazawa University (114132‐1).

## Consent

Written informed consent was obtained from the patients for the publication of this case series, including any accompanying images or data.

## Conflicts of Interest

The authors declare no conflicts of interest.

## Supporting information



Supporting information

## Data Availability

Further data including summaries of systemic therapies to URCA, patients’ information, and histological images are available at Figshare (10.6084/m9.figshare.30560060).
