# Supplementary material for: Remarkable Responses to Chemoimmunotherapy in Ultra‐Rare Urachal Cancer: A Case Series and Reverse Translational Research
Source: MedComm (2020). 2025 Dec 8;6(12):e70516. doi: 10.1002/mco2.70516 (PMC12686130; doi:10.1002/mco2.70516)
Supplement: Supplementary file 1 — Supporting information [file MCO2-6-e70516-s001.docx]

Supplementary Information for

Remarkable Responses to Chemoimmunotherapy in Ultra-Rare Urachal Cancer:

A Case Series and Reverse Translational Research

Hiroshi Kotani, Shigeki Sato, Kazuyoshi Shigehara, Daisuke Saito, Hiroyuki Sakaguchi,

Akihiro Nishiyama, Isao Matsumoto, Atsushi Mizokami, Seiji Yano, and Hiroaki Taniguchi

Correspondence to: h.kotani@staff.kanazawa-u.ac.jp

**This file includes:**

Materials and Methods

**Materials and methods**

**Overview of Urachal cancer**

Urachal cancer (URCA) is a primary adenocarcinoma arising from the urachal remnant. URCA is typically located at the bladder dome or anterior wall and connects to the urachus. The histopathological subtypes are classified as adenocarcinoma (90%: often mucinous/enteric-type, or signet-ring cell) or non-glandular carcinoma (Rare: urothelial carcinoma, squamous cell carcinoma, small cell carcinoma). Signet-ring cell morphology often leads to poor prognosis. URCAs frequently harbor *KRAS*, *TP53*, and *GNAS* mutations and mostly show microsatellite stability.

**Participants**

Patients had histologically confirmed unresectable or relapsed URCA, were in good general condition (Eastern Cooperative Oncology Group performance status of 0 or 1), and had adequate organ functions for SOX/nivo prior to treatment initiation. HER2 and PD-L1 expression in tumor specimens was evaluated by immunohistochemistry. Sheldon staging was used for staging because URCA develops from urachal residues seen mainly in the midline or the wall of the bladder, and the tumor-node-metastasis criteria for cancers in the bladder are not appropriate for URCA (1). For assessment of therapeutic responses, the Response Evaluation Criteria in Solid Tumors guidelines, version 1.1 (RECIST 1.1) criteria were used.

**Treatment and follow-up**

The SOX/nivo regimen consisted of chemotherapy plus an anti-PD-1 antibody every 3 weeks (oral S-1 40 mg/m^2^ twice daily on days 1-14, intravenous oxaliplatin 130 mg/m^2^ on day 1, in addition to intravenous nivolumab 360 mg/body on day 1). The dosing schedule was adjusted for individual patients in clinical practice. The lesions were assessed and monitored by contrast-enhanced CT or FDG PET/CT as the baseline and evaluated every two cycles for the first six cycles and then at appropriate time points.

**Genetic testing**

Comprehensive genetic testing was performed on tumor and blood samples obtained prior to the initiation of SOX/nivo, under the MONSTAR-SCREEN-2 protocol following informed consent. DNA and RNA extracted from the samples were analyzed via the MI Exome whole exome sequencing (WES)/the MI Transcriptome whole transcriptome sequencing (WTS) at Caris Life Sciences. The MSI status and TMB were also evaluated. Blood samples were used as a reference for germline DNA testing and circulating tumor DNA analysis. Significant genetic alterations identified in tumor samples were as follows: Case 1; *ATM 6807+2T>C, KRAS Q61R, RAD54L V411fs*: Case 2; *ERBB2 G776V, KMT2C P3015fs, NF1 R2258*, RB1 R579*, RNF43 G754fs, TP53 R175*: Case 3; *AKT2 amplified, APC S1465fs, ARID2 Q1031*, KIT V50M, TP53 Q167**: Case 4; *GNAS R201H, KMT2C c.3499+1_3499+7del7, MYC amplified, TP53 R273H*. The significant results of blood samples were followed; Case 1; none: Case 2; none: Case 3; *APC S1465fs, ARID1A R1276*, ARID2 Q1031**: Case 4; none. No classified gene fusions or transcript variants were detected in any of the samples by WTS.

**Reagents**

5-FU and L-OHP were obtained from MedChemExpress. RPMI 1640, fetal bovine serum, and penicillin/streptomycin were obtained from Gibco.

**Cells**

MISB18 cell line established from an URCA patient who received multiple lines of chemotherapies was obtained from Misvik Biology Ltd (2). Cells were cultured in RPMI 1640 supplemented with 10% heat-inactivated fetal bovine serum, 100 U/mL penicillin, and 100 μg/mL streptomycin. The cell number and viability were determined using a Countess II FL Automated Cell Counter (Thermo Fisher Scientific). The controls were treated with 0.1% DMSO.

**Cell viability assay**

Cells were seeded in 96-well plates so that the control cells reached approximately 80% confluency at the end of the assay. The next day, cells were treated with the indicated compounds or 0.1% DMSO (n = 6). After 72 hours, the Cell Counting Kit-8 (Dojindo) was added to the cells, and cell viability was assessed by measuring absorbance. The nonlinear regression model with a sigmoidal dose-response curve was visualized using GraphPad Prism 9. Combined drug effects were analyzed using SynergyFinder (<https://synergyfinder.org/#!/>).

**Immunoblot analysis**

Cell lysates were collected using CelLytic M (Sigma-Aldrich) supplemented with 1% phosphatase inhibitor cocktail 3 (Sigma-Aldrich) and 10 μM phenylmethanesulfonyl fluoride (Sigma-Aldrich). Immunodetection of proteins was performed using standard protocols. Cleaved-PARP, calreticulin, and GAPDH antibodies were obtained from Cell Signaling Technologies. Signals were detected using a Chemiluminescence Imaging System (M&S Instruments Inc.).

**References**

1. Sheldon CA, Clayman RV, Gonzalez R, Williams RD, Fraley EE. Malignant urachal lesions. *J Urol*. 1984;131(1):1-8.
2. Mäkelä R, Arjonen A, Härmä V, Rintanen N, Paasonen L, Paprotka T, et al. Ex vivo modelling of drug efficacy in a rare metastatic urachal carcinoma. *BMC Cancer*. 2020;20(1):590.
